# A New Genre of Digital Texts That Explore Children’s Frame of Mind, Health Literacy Skills, and Behavioral Intentions for Obesity Prevention

**DOI:** 10.3390/children12060663

**Published:** 2025-05-22

**Authors:** Valerie A. Ubbes

**Affiliations:** Department of Kinesiology, Nutrition and Health, Miami University, Oxford, OH 45056, USA; ubbesva@miamioh.edu; Tel.: +1-513-805-6109

**Keywords:** obesogenic body frame, frame of mind, Habits of Health and Habits of Mind© model, functional health literacy, interactive health literacy, health literacy event, constructivist pedagogy, writing, digital texts, health literacy narrative, multimodal, obesity prevention

## Abstract

**Background:** This project focuses on the relevance of using a health literacy approach to educating children about obesity prevention. The Habits of Health and Habits of Mind© model was used to write Electronic Texts for Health Literacy© to encourage actions that support obesity prevention. Guided by the Integrative Theory of Behavioral Prediction, the design template for a new genre of digital texts called Electronic Texts for Health Literacy© emerges for exploring children’s frame of mind, health literacy skills, and behavioral intentions toward obesity prevention. **Methods**: Online materials from selected websites were strategically reviewed for improving obesity prevention and child health literacy. The digital resources were juxtaposed with the Electronic Texts for Health Literacy©, with the latter written by and for children. **Discussion**: Health educators who use a constructivist pedagogy can help students to write health literacy narratives about obesity prevention, then read and talk about their multimodal compositions to further the practice and development of their health literacy skills. Children with obesogenic body frames can also gain from cowriting visual–textual–gestural health narratives with their peers or health professionals. Co-constructed narratives can help children make deeper connections about their identity, frame of mind, and social agency. **Summary**: Although this untested resource is available as a new genre of digital text, health educators could nudge children toward developing a stronger frame of mind and behavioral intentions toward obesity prevention when they write health literacy narratives that focus on decision making, goal setting, and communication in the context of eating nutritious foods and participating in physical activities.

## 1. Introduction

The U.S. Preventive Services Task Force [1] has issued updated intervention guidelines for children who are obese. Current data show that 21–24 percent of American children and adolescents are overweight, and another 16–18 percent are obese [2]. Obesity is the most prevalent nutritional disorder among children and adolescents in the United States [3]. The World Health Organization has indicated that global mortality from overweight and obesity conditions is more deadly than underweight conditions. The main health consequences of overweight and obesity are heart disease, stroke, osteoarthritis, cancers, type 2 diabetes, and sleep apnea [4], including childhood nonalcoholic fatty liver disease [5]. In 2023, 41 million children under the age of five were overweight, representing nearly six percent of this age group. Globally, over 340 million children and adolescents aged 5 to 19 are overweight, representing nearly 18 percent of this age group [6]. During the coronavirus pandemic, children’s health routines were disrupted, which affected their motor skill development [7], eating and physical activity behaviors [8], and lowered their metabolism toward obesity and weight gain [9]. Obesity has also been associated with chronic low-grade inflammation that accelerates the aging process and increases the risk of cognitive decline [10].

Children with large body compositions may have compromised oral communication skills and lower linguistic abilities due to increased shortness of breath, being more easily fatigued, and having a hoarse throat with an altered sound to their voices (https://www.greatspeech.com/can-weight-gain-cause-speech-problems/ (accessed on 3 March 2025)). In addition to physiological concerns, children with obesogenic frames require support for psychosocial and emotional development. Children who lack social role models for healthy eating and physical activity may also lack development in their pro-social oral language and cognitive–motor literacy skills. Educators and parents can help all children acquire multimodal language and literacy development [11]. A child with underdeveloped speech and/or delayed language patterns may not know how to negotiate hurtful comments or disrespectful looks regarding their obesogenic body frame. Building up a child’s emotional core will influence their attitude or behavior, known as their frame of mind. Children may lack physiological and psychological motivation [12], given that there may be a psychosocial burden of living with a visible disfigurement and stigma [13]. Stigma is defined as the devaluation of social identities based on the recognized difference of a distinguishing characteristic from one’s peers [14].

A recent area of concern in obesity prevention is oral health hygiene. Oral health hygiene is integral to general health and is associated with eating fresh foods and nutritious beverages. The gut biome is responsible for regulating and balancing the lymphatics of the immune system in disease prevention. According to the World Health Organization [15], a key health-promoting message is that “Oral health is the state of the mouth, teeth and orofacial structures that enables individuals to perform essential functions such as eating, breathing and speaking, and encompasses psychosocial dimensions such as self-confidence, well-being and the ability to socialize and work without pain, discomfort and embarrassment”. When children do not brush and floss their teeth adequately and consistently, opportunistic diseases can occur, many with connections to overweight conditions and obesity [16]. Oral health literacy is especially susceptible to literacy interventions because narratives and scripted messages can incorporate valid and reliable health information, discuss health products such as toothpaste and toothbrushes, and promote health services such as visiting the dentist for a six-month checkup [17].

Many children grow up learning health-related routines that are foundational to life. Children should be exposed to health-related messages when they are also learning and practicing health behaviors. By reading to and reading with children, the language of health can extend to the lifestyle practices of the home and school. Both home and school environments should surround children with messages of health. Valid and reliable health information can be shared in conversations and vetted through print and electronic resources to augment what is exchanged during interpersonal communication [18]. Other ways to ensure that the information can be accessible for the greatest number of people are to use multimodal language approaches [19,20] and print and electronic multimedia approaches [21]. In a systematic review of 12 empirical studies looking at health literacy in early childhood, Banfai-Csonka and colleagues [22] found that picture-based messages or story-based messages with illustrations helped to support the development of health literacy when the studies focused on food, nutrition, and oral health. Children’s picture books that are used to promote health-related concepts, topics, and skills have been promoted for decades [21].

Beyond picture books for early childhood years, there are informational websites about obesity prevention that cater to children and youth. For example, the United States Department of Health and Human Services promotes a GirlsHealth.gov site, https://girlshealth.gov/nutrition/healthyweight/index.html#obesity (accessed on 3 March 2025) with a subtitle called “Be Healthy. Be Happy. Be You. Beautiful”. Valid and reliable health information is shared on nutrition and physical activity, with short personal testimonials from two youth who are both challenged and satisfied with their body weight. Another resource for girls, boys, and their parents is found at TeensHealth.org, https://kidshealth.org/en/teens/obesity.html (accessed on 3 March 2025), which is a Kids Health initiative that offers a scientific range of health topics, including overweight, obesity, metabolic syndrome, and insulin resistance. The narrative information is objective, factual, and written at an adult level, similar to the HealthforKids.co.org website for parents from the United Kingdom’s National Health Service. However, the latter also includes interactive games and activities highlighted by avatars to teach children and youth about their health, especially healthy eating and keeping active to prevent diabetes. The narratives are written directly to children with no direct information on obesity or overweight unless tied to a disorder or disease. One website written for early childhood years into adulthood is the MyPlate.gov website (https://www.myplate.gov/, (accessed on 3 March 2025)) from the United States Department of Agriculture. Child-friendly games and apps are supported by songs, posters, recipes, and videos with anthropomorphized food characters teaching about food. This science-based content is interactive for children, youth, and adults while encouraging them to eat nutritious foods and to lead an active lifestyle.

Opportunities to boost functional health knowledge and health-related skills around obesity prevention can be planned with guidance from the National Health Education Standards. With its focus on accessing valid and reliable health information, products, and services [18], teachers can plan and implement functional health literacy events with children [23] to build functional health literacy [18]. Health educators can also implement effective curricula through role plays, group cooperation, and active learning strategies using more advanced technologies [24], such as those used in the websites named above. Health educators need to promote the benefits of literacy-rich environments where children read, write, and speak daily about their health during their developmental years while also practicing interactive health literacy skills when communicating with their peers, parents, and health professionals using health-related skills and behaviors [25].

The purpose of this paper was to describe a new innovative strategy for improving health literacy skills through a new genre of health literacy texts that explore children’s frame of mind and behavioral intentions toward obesity prevention. Electronic Texts for Health Literacy© was designed to capture the vocabulary and language that children need when learning to be healthy. The texts depicted in this paper are tailored to younger elementary and middle school-aged children who write about their thoughts and feelings toward health behaviors and social norms that support obesity prevention. The cognitive behavioral skills outlined in the Habits of Health and Habits of Mind Model© can support children to recognize the relationship between their daily health habits and quality of life [25]. Specifically, children gain the ability to demonstrate health literacy when they read, write, and speak about their Electronic Texts for Health Literacy©, with a focus on the cognitive skills of goal setting, decision making, or communication. Many of the Electronic Texts for Health Literacy© use one or more Habits of Health (i.e., behaviors) and one Habit of Mind (i.e., cognitive skill) to model how people can be healthy on a daily basis. As children write and talk about their functional health knowledge and salient beliefs, they will be demonstrating how to translate decision making, goal setting, and communication skills into health behaviors for obesity prevention.

For the purposes of this project, health literacy events were adopted from the work of Rumenapp and colleagues [23] (p. 11), who conceptualized them as “the ways in which students, teachers and other community members can use a focus on health literacy to accomplish particular health goals”. In the current project, when students were encouraged to use functional health literacy skills to write health narratives about obesity prevention, the literacy event of writing an Electronic Text for Health Literacy© was focused on meeting a personal health goal. The value of that health literacy event within a health education classroom setting was to generate a variety of Electronic Texts for Health Literacy© through the lived experiences of children and specifically [26] to affect educational outcomes and school achievement among children who are obese. Therefore, our methodology is both conceptual and illustrative.

## 2. The Health Literacy Database at Miami University

This section highlights the Health Literacy Database at Miami University, which is a tool for locating digital materials on a variety of health topics that are valid, reliable, authentic, and equitable for children, youth, and adults [21]. A search for the number of Electronic Texts for Health Literacy© resulted in 98 health narratives on physical activity, 72 health narratives on nutrition, and 55 health narratives on relationships, which are important as strategies for obesity prevention. From a total collection of 190 digital text compositions, 52 percent (n = 98) focused on physical activity, 38 percent (n = 72) focused on nutrition, and 29 percent (n = 55) focused on relationships. A search for the term “obesity” produced only three Electronic Texts for Health Literacy©, which represented only one percent (n = 2) of the available multimodal health narratives. As such, selected narratives are summarized below to provide examples of valid and reliable health information from which obesity can be prevented or reduced from a child-centric or youth-centric perspective. 

**Figure 1:**
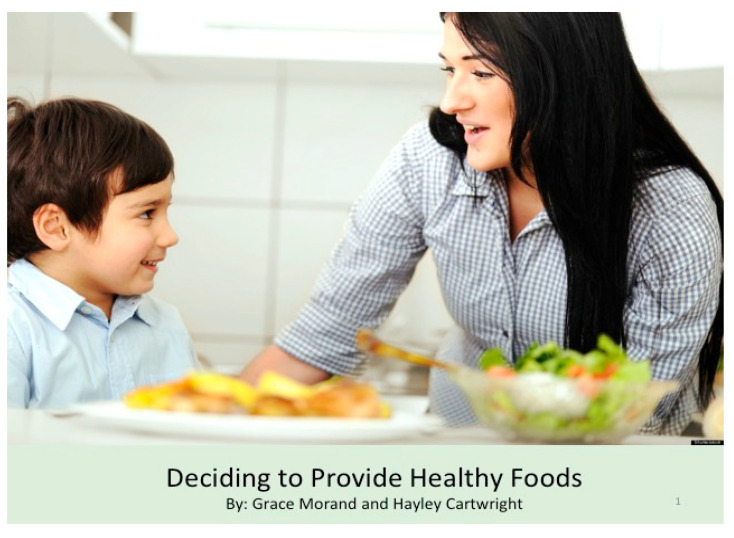
Example of an Electronic Text for Health Literacy© title page demonstrating one Habit of Health (nutritious foods) and one Habit of Mind (decision making).

**Figure 2:**
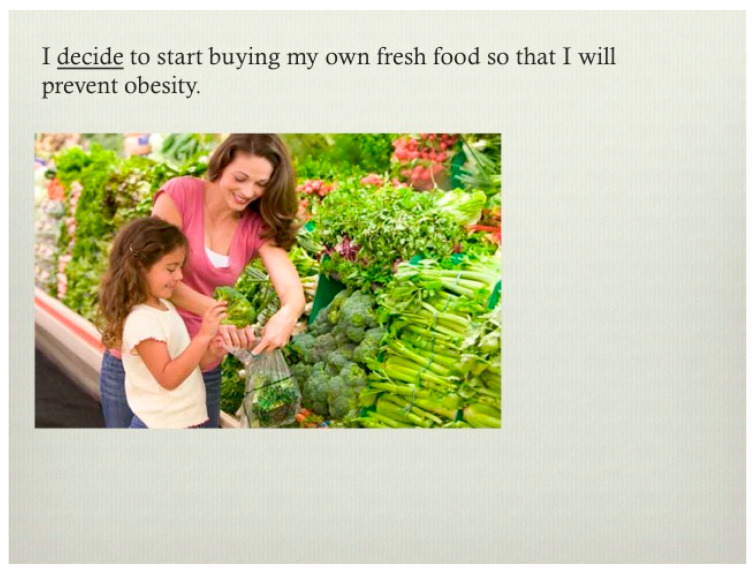
Example of a health literacy narrative demonstrating one Habit of Health (obesity prevention) and one Habit of Mind (decision making) in a grocery store.

Figure 1 and Figure 2 show examples of a title page and a narrative slide, respectively, from an Electronic Text for Health Literacy©. The main design features of this new genre include an authentic human photograph depicting social norms for health, supported by words that are emphasized by underlining the cognitive verbs (e.g., deciding, decide, setting goal, and goal setting). Another design feature includes the child’s voice in the first-person narrative, written by the child, so that the child learns to identify and value the importance of healthy eating and physical activity behaviors for obesity prevention. On the website, keywords are listed to highlight ways to search for that particular Electronic Text for Health Literacy©, including which salient beliefs and which National Health Education Standard(s) are aligned to the visual–textual–gestural narrative.

**Figure 3:**
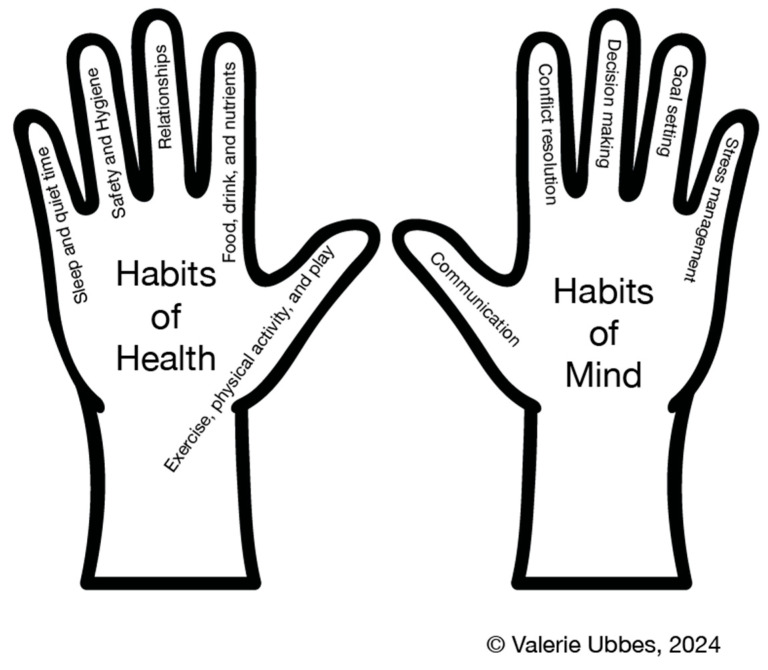
Habits of Health and Habits of Mind© model.

Children are able to learn vocabulary for writing and talking about health when they use the Habits of Health and Habits of Mind© model to write an Electronic Text for Health Literacy©. Figure 3 shows the HH&HM model below. Students choose one Habit of Health (a health behavior) and one Habit of Mind (a cognitive skill) when writing an Electronic Text for Health Literacy©.

Health education is a social and behavioral science that communicates how individuals and groups of people can build cognitive behavioral skills through continual practice [25]. The theoretical framework used when writing the Electronic Texts for Health Literacy© is the Integrative Theory of Behavioral Prediction [27], which has been described elsewhere [17]. Table 1 shows an example of a health literacy narrative that focuses on nutrition and decision making while addressing three components of the Integrative Theory of Behavioral Prediction, e.g., efficacy beliefs, health outcome beliefs, and normative beliefs. The transcript in the right column of Table 1 shows how the text of the health literacy narrative entitled “Making the Decision to Eat Fresh Food” is aligned with the three beliefs that are listed in the left column. The “I believe” and “I decide” statements are examples of how the word patterns in the design template focus on repeating the practice of health-related language and skill development, which has the potential to lead to healthy behaviors.

**Figure 4:**
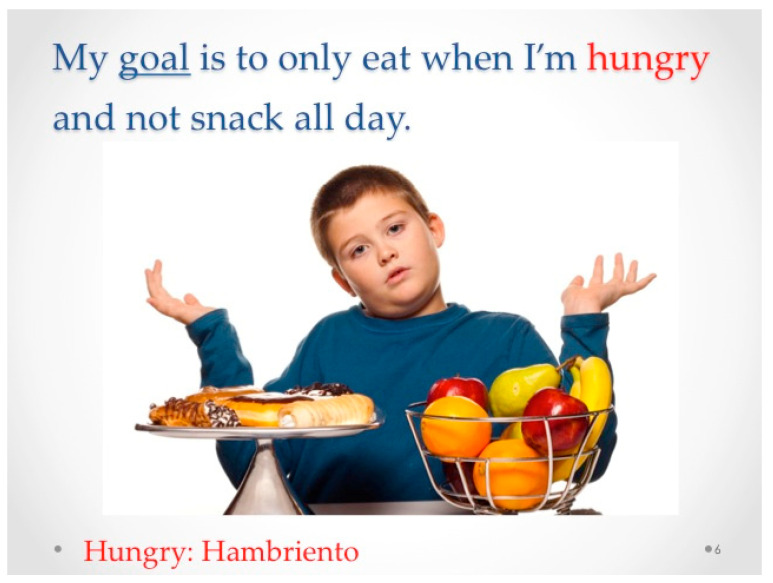
Example of a health literacy narrative for goal setting and nutrition (in both English and Spanish).

**Figure 5:**
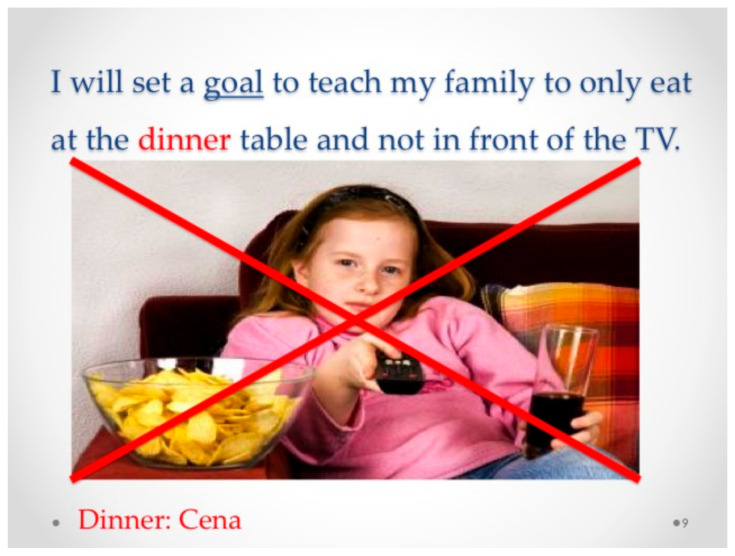
Example of a loss frame strategy in a health literacy narrative for goal setting and nutrition at home (in both English and Spanish).

**Figure 6:**
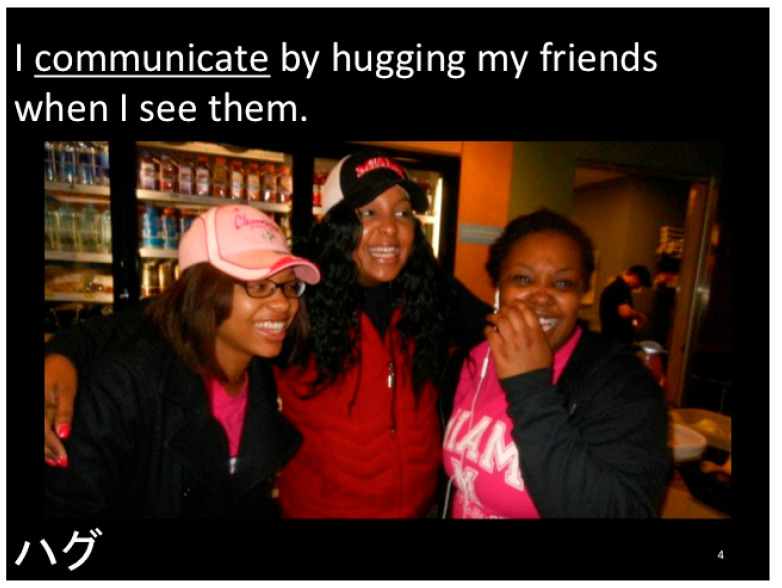
Example of a health literacy narrative for communication and relationships, including social norms from friends (peers) in a food and beverage market.

**Figure 7:**
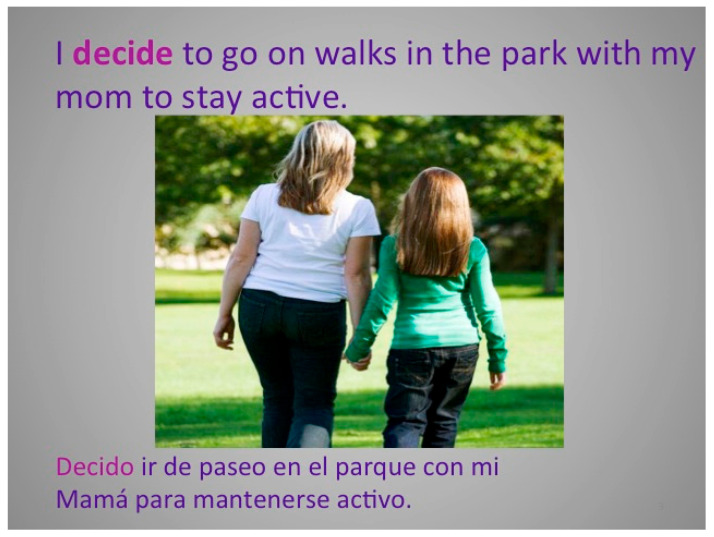
Example of a health literacy narrative for decision making, relationships, and physical activity (bilingual), including social norms from a parent in nature.

**Figure 8:**
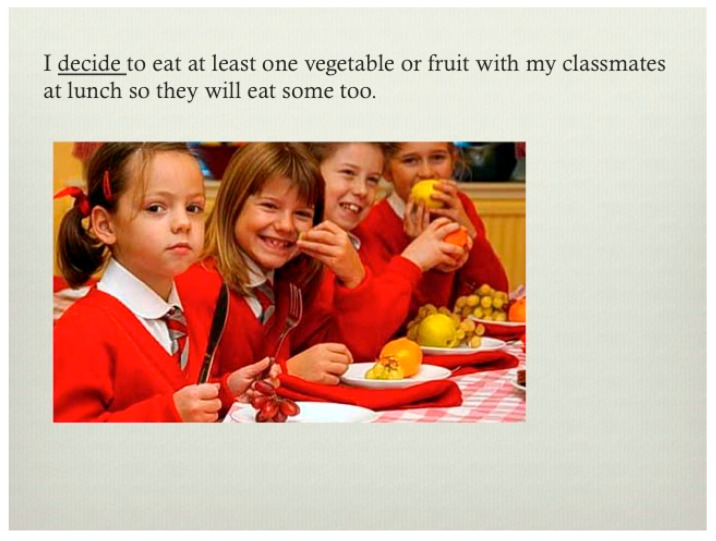
Example of a health literacy narrative for decision making and nutrition, including social norms from peers at school.

**Figure 9:**
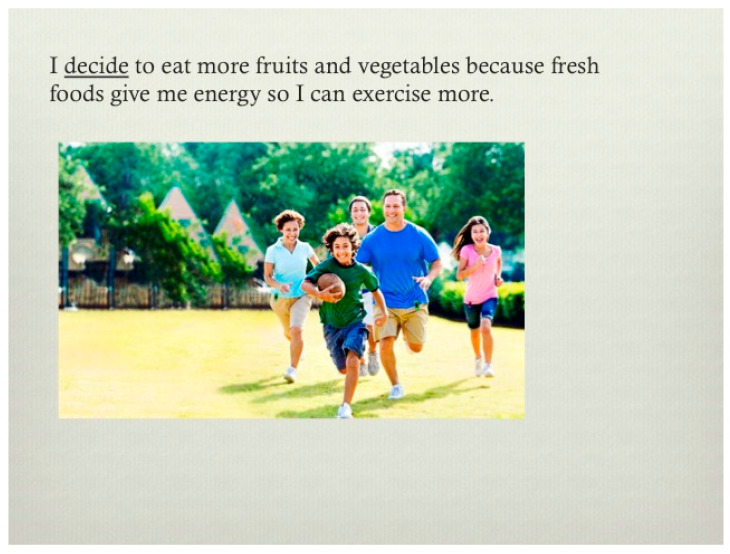
Example of a gain frame strategy for a health literacy narrative for decision making, nutrition, physical activity, and relationships, including social norms from a family playing outdoors.

**Figure 10:**
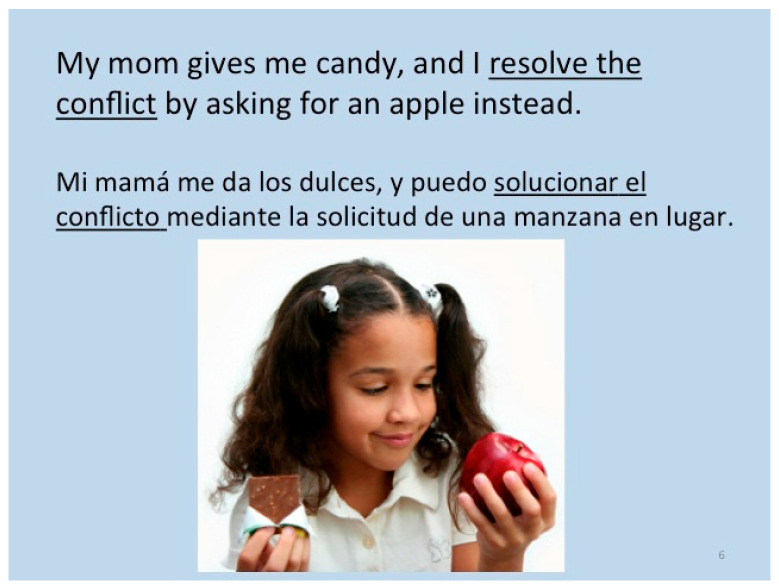
Example of a gain frame strategy in a health literacy narrative for conflict resolution and nutrition (bilingual).

Figure 4, Figure 5, Figure 6, Figure 7, Figure 8, Figure 9 and Figure 10 show sample pages from eight different Electronic Texts for Health Literacy© cowritten by the principal investigator (PI) and students from Miami University (Oxford, OH, USA) who had academic majors in health education, elementary education, or public health, with future plans to work with children. The common lexical feature of the design template from the PI was declarative sentences that emphasized an active verb employing one Habit of Mind skill, e.g., decision making, goal setting, or communicating, and one or more Habit of Health behaviors, e.g., nutrition, physical activity, or relationships, for obesity prevention. Each narrative was written to include a sequence of three theoretical beliefs within the ten-page composition (See Table 1). The writer-narrator also used historical thinking when describing “what, where, why, when, and with whom” the children were doing reasoned actions for disease prevention. The visual, textual, and gestural design template promoted multimodal cues for action [28] and gave students practice in writing, reading, and speaking about their compositions with others to enhance their functional and interactive health literacy skills.

In each of the examples below, the HH&HM model is shown to demonstrate actions for health. Two of the health literacy narratives focus on goal setting, three focus on decision making, one focuses on communication, and one focuses on conflict resolution. Please note that each narrative is removed from its ten-slide composition to provide a variety of examples. Please visit the website at https://dlp.lib.miamioh.edu/healthliteracy/ (accessed on 3 March 2025) to see each of these separate examples intact in their original compositions.

## 3. Recommendations

In the current study, narratives about obesity prevention included one Habit of Health (behavior) and one Habit of Mind (cognitive skill). Specifically, the Habits of Health topics of nutrition, physical activity, and/or social relationships were combined with the Habits of Mind skills of decision making, goal setting, and communication. Narratives employed an arts-based methodology, which used a photographic-based inquiry as a social documentary [29]. Even though the “lived experiences” of the students as writers included a few selections of photos with obesogenic frame sizes, Figure 5, Figure 9 and Figure 10 shown above indicated that student writers were also aware of gain frame and loss frame concepts when they composed their health literacy narratives via the design template provided by the PI. Gain frame message design was defined as a positive message that encourages an action or behavior, whereas loss frame message design referred to the risks in not doing an action or behavior [30].

When working with the psychological aspects of obesity, educators and researchers need to support children and youth as they discover the underlying beliefs, motivations, and realities of their obesity. Health programs and educational materials should address the three salient social norms (i.e., normative beliefs, health outcome beliefs, and self-efficacy beliefs) surrounding healthy and unhealthy decisions [27]. Creating new resources, such as an Electronic Text for Health Literacy©, gives young people a chance to compose narratives that are relatable and authentic for themselves and others. Semi-structured interviews are useful for learning about the needs and perspectives of children and youth. Schunk and DiBenedetto [31] emphasized that self-efficacy was domain-specific and that a general self-efficacy does not exist. Therefore, research focusing on cultural contexts is important since non-Western cultures tend to place greater emphasis on effort as a reason for success, whereas Western cultures emphasize ability. By involving children in writing health literacy narratives and choosing what cognitive skill they will employ when working toward a health behavior, there is a greater chance that their narrative constructions will draw from their sociocultural backgrounds. Health education teachers can use the components, elements, and examples in Table 2 (below) when organizing the writing of an Electronic Text for Health Literacy© to ensure fidelity to the design framework.

One important educational goal for children and youth is to promote the need for daily selfcare routines that lead to health habits. As a “hands-on” concrete visual, the HH&HM model becomes a practical tool for supporting the development of health-related patterns and daily routines. As children and youth mature, the importance of teaching them how to set boundaries to maintain or improve their health is also valuable. Boundaries can be described as frames of mind that are something you “set” or “make” based on a plan of action, such as a health literacy narrative. School health education classes can use health literacy narratives with young people to advance their frames of mind on prevention. Health literacy events [23] can also be incorporated into other academic courses, e.g., language arts and social studies. For example, teachers can encourage a deeper reading of an Electronic Text for Health Literacy© by talking about and interpreting a variety of narratives on a social level (i.e., family and community) and a personal level (i.e., individual), which fosters students’ ability to understand how their decision making and goal setting actions for health can be affected by different contexts and environments.

As a pedagogical tool, teachers can ask students to write an electronic text on obesity prevention with a peer to gain multiple perspectives as they compose a multimodal health literacy narrative together in words, pictures, numbers, gestures, and environmental cues. Teachers can also ask students to compose several health literacy texts for obesity prevention using different cognitive skills (e.g., decision making, goal setting, and communication) with different age groups and cultural backgrounds. One motivation behind the constructivist pedagogical approach is that students have become too reliant on technology, and “have become disempowered, giving power to their digital devices, and not relying on the power within themselves” [32]. Because Keator [32] suggests that students are “Fixated on their digital devices, (and) they no longer have the capacity to be self-aware, aware of the needs of others, engage a literary text or relate to their surrounding environment”, a health literacy project such as the one outlined in this paper engages students to use their computers and digital devices in a proactive way as they become health-educated thinkers and actors toward their daily health choices, goals, and behavioral intentions.

The National Health Education Standards were written to guide the development of curricula and instruction for preK–12 health education [18]. Eight national standard statements promote student knowledge and skill development for health education. One of the eight standards focuses on health literacy, which reads “Students demonstrate health literacy by accessing valid and reliable health information, products, and services to enhance health”. The word “valid” refers to health information that is accurate, credible, and not misleading [18]. The word “reliable” means consistent and trustworthy [18]. Health literacy can include functional health literacy, which is a personal skill, and interactive health literacy, which is an interpersonal social skill. These two forms of health literacy are organized by grade level to be developmentally appropriate across the preK–12 school years [18]; however, the original framework lacks the third component, critical health literacy [33]. By demonstrating functional and interactive health literacy, students will gain multiple ways to access valid and reliable health information, products, and services to enhance their health during health instruction [18]. This paper also suggests that students should be reading, writing, and speaking about health information, products, and services with others.

In research with young children who were given the opportunity to speak for themselves, Derwig, Tiberg, and Hallstrom [34] determined that children could take an active role in their health by interpreting illustrated health messages in a different way than adults. Whereas social scientists seek the “social and cultural practices and processes” [35] (p. 143) embedded in photographs, Mitchell and Allnutt [29] suggested that photographs can be representative of change and may frame the “representational agents of change” (p. 259). In fact, any photographs that children take and/or choose for their digital texts may “serve to give voice” (p. 259). Child-centered constructivist approaches are important in health education [25], which includes the use of age-related and developmentally related approaches for health literacy, especially when distinguishing school children and adolescents from adults [36,37] and when transitioning preschool children into elementary and secondary schooling [38]. According to the National Health Education Standards [18], some of the performance expectations for grade 2 students would be “to use functional health literacy (e.g., reading, writing, and speaking) to access trustworthy health information to learn functional health knowledge”, and then by grade 5 “to use functional health literacy skills (e.g., reading, writing, and speaking) to access valid and reliable health information to learn about health behaviors”. Another grade 5 expectation would be to “access multimodal health messages (e.g., words, pictures, numbers, and/or gestures) in print or electronic materials to practice interactive health literacy”. By grade 8, students would “access credible websites or health-related applications using technology to support health behaviors” and then by grade 12, students would “demonstrate functional health literacy (e.g., reading, writing, and speaking) to evaluate valid and reliable health information about a health behavior” [18].

Vygotsky’s theory of constructivism [39] emphasized that children who had social interactions with adults and more capable peers helped to scaffold their learning through the Zone of Proximal Development into constructions of meaning or understanding. Echoing Vygotsky’s emphases on the importance of language at the center of learning and language being a tool of culture, Gavelek and Raphael [40] (p. 183) stated that language mediates “the relationship between the student as knower and the text as known; both the construction of what comes to be understood as knowledge of a text and development of student as knower of texts depends upon language practices”. This meets the stated purpose of this project, which used the HH&HM model so that children can learn a vocabulary for reading, writing, and talking about their health through compositions that promote health literacy as a strategy for obesity prevention. Student assessments might go beyond simply writing or cowriting an electronic text as a culminating health literacy event. Students can also read a wide variety of health literacy narratives that their peers have written and provide feedback on any aspect of the visual–textual–gestural messages for obesity prevention, including the extent to which the scripts were convincing and motivating to them using a numerical rating scale. Asking students to write a brief paragraph on how their understanding of obesity prevention has changed from the start to the end of the health literacy project is another assessment that aims to capture the salient beliefs and behavioral intentions toward eating nutritious foods and participating in physical activity as Habits of Health.

Recommendations for future research should consider how counselors, therapists, and social workers might converse and collaborate with children and youth who have obesity or overweight conditions. The ability to write something together while talking things out lends itself to identity formation and social agency through health literacy. As children and youth become empowered by their oral and written self-talk, clinicians can encourage them to frame their thinking even more by practicing the writing of cognitive skills (e.g., Habits of Mind) and by selecting photography that depicts behavioral intentions for obesity prevention (e.g., Habits of Health). Beyond one empirical study on the HH&HM Model [41], there is a need for more empirical studies on the model, including the need to compare the utility of the HH&HM model as an effective approach in the design of health literacy narratives beyond the “usual care” approaches for obesity control. Once additional digital texts are written that use health literacy skills to explore children’s frame of mind and behavioral intentions toward obesity prevention, future researchers could use assessment tools such as the PEMAT [42] to study the extent to which the educational material is actionable with students who have varying levels of health literacy. In addition, the actionability and behavioral intentions associated with the written design template, which specifically highlights efficacy beliefs, health outcome beliefs, and normative beliefs, should be empirically tested through a valid and reliable health literacy instrument.

## 4. Conclusions

This project focused on the relevance of using a health literacy approach to educate children about obesity prevention. Selected digital materials for obesity prevention were reviewed for improving child health literacy. The online resources were juxtaposed with a new genre of digital texts called Electronic Texts for Health Literacy©, which explored children’s frame of mind, health literacy skills, and behavioral intentions toward obesity prevention. A Habits of Health and Habits of Mind© model was used as a design template for constructing multimodal health narratives so that children could use a health vocabulary for reading, writing, and speaking about obesity prevention. Through a constructivist pedagogy, health educators and classroom teachers can nudge children toward developing a frame of mind and behavioral intentions toward obesity prevention when they write health literacy narratives that focus on decision making, goal setting, and communication in the context of eating nutritious foods and participating in physical activities.

## Figures and Tables

**Table 1:** Example of a health literacy narrative with three theoretical components.

Theoretical Components	Transcript of a Health Literacy Narrative
Skill: Decision Making	Making the Decision to Eat Fresh Food by Melanie Dow https://dlp.lib.miamioh.edu/healthliteracy/items/show/298 (accessed on 3 March 2025)
Efficacy Belief	I believe that I will make the decision to eat more fruits and vegetables at a young age because fresh food makes me live longer.
Health Outcome Belief	I decide to eat more fruits and vegetables because fresh foods give me energy so I can exercise more.
Health Outcome Belief	I decide to start buying my own fresh food so that I will prevent obesity.
Health Outcome Belief	I decide to start eating more fruits and vegetables because fresh foods can strengthen my immune system and prevent illnesses.
Normative Belief	I decide to encourage my family to start snacking on fresh food so that we will have a reduced risk for heart disease.
Normative Belief	I decide to eat at least one vegetable or fruit with my classmates at lunch so they will eat some too.
Health Outcome Belief	I decide to eat a fruit instead of a dessert after dinner in order to get my daily nutrient and vitamin intake.
Efficacy Belief	I believe that my decision to eat more fresh foods at meals will help me to live a healthier lifestyle.
Interactive Health Literacy	Summary: I’ve made the decision to eat fresh food. How about you? Have you made a decision too?

**Table 2:** Design framework of Electronic Texts for Health Literacy©.

Design Framework	Components	Elements	Specific Examples
Conceptual	Habits of Health and Habits of Mind Model	Health Behaviors as Habits of Health	
			Food, Nutrition, and Beverages
			Physical Activity
			Relationships
			Sleep and Rest
			Safety and Hygiene
		Cognitive Skills as Habits of Mind	
			Decision Making
			Goal Setting
			Communication
			Conflict Resolution
			Stress Management
Theoretical	Integrative Theory of Behavioral Prediction	Salient Beliefs	
			Efficacy Beliefs
			Health Outcome Beliefs
			Normative Beliefs
	Constructivist Theory	Language is used as a tool for making meaning. Learning is socially constructed.	
			Written Language
			Oral Language
			Body Language
			Multimodal Language
Narrative	Health Literacy Narrative		
		Visual	
		Textual	
		Gestural	
Framing	Gain Frame Message		
	Loss Frame Message		

## Data Availability

Links to publicly archived datasets are available at the Digital Literacy Partnership website @ https://dlp.lib.miamioh.edu (accessed 3 March 2025). Specific resources shared in this manuscript are available from the Health Literacy Database at Miami University @ https://dlp.lib.miamioh.edu/healthliteracy/ (accessed 3 March 2025).
